# Four Sets of Incisors

**Published:** 1859-11

**Authors:** 


### FOUR SETS OF INCISORS.

Examined to-day a case in the mouth of Mr. A. A. S, of New York City. There has been in the case rather an unusual developement of teeth, especially so far as the superior incisors are concerned. At six years of age the deciduous incisors were shed as usual. The permanent teeth were erupted in the usual manner. At eleven years of age the superior incisors were so much decayed as to render their removal necessary, at the age of sixteen, a third set of firm, strong incisors were erupted, these remained till the age of thirty-nine years, when in consequence of decay, they had to be removed; they were very strong firmly attached teeth, requiring great force for their removal. A rubber job was made and put in about three weeks after the removal, they have now been worn two weeks, and at this time, there is a central incisor of the fourth set being erupted, the edge about one-eighth of an inch through the gum, it prevents the plate from taking hold upon the arch. The edge of the tooth, is that of a well defined central incisor, and in proper position.

It is strange that within six weeks of the removal of the third set of incisors, all firm and full grown roots, that one of the fourth set should appear. Its growth is very rapid. Will any one give the physiological explanation? T.

